# Molecular Mechanisms Elicited by d-Aspartate in Leydig Cells and Spermatogonia

**DOI:** 10.3390/ijms17071127

**Published:** 2016-07-14

**Authors:** Maria Maddalena Di Fiore, Alessandra Santillo, Sara Falvo, Salvatore Longobardi, Gabriella Chieffi Baccari

**Affiliations:** 1 Dipartimento di Scienze e Tecnologie Ambientali, Biologiche e Farmaceutiche, Seconda Università di Napoli, Via Vivaldi 43, 81100 Caserta, Italy; Alessandra.Santillo@unina2.it (A.S.); Sara.Falvo@yahoo.it (S.F.); Gabriella.Chieffi@unina2.it (G.C.B.); 2 Medical Affairs Fertility, Merck KGaA, Frankfurter Straße 250, 64293 Darmstadt, Germany; salvatore.longobardi@merckgroup.com

**Keywords:** d-aspartate, steroidogenesis, spermatogenesis, spermatogonia, Leydig cells, NMDAR, MAPK, STAR, PCNA, AURKB

## Abstract

A bulk of evidence suggests that d-aspartate (d-Asp) regulates steroidogenesis and spermatogenesis in vertebrate testes. This review article focuses on intracellular signaling mechanisms elicited by d-Asp possibly via binding to the *N*-methyl-d-aspartate receptor (NMDAR) in both Leydig cells, and spermatogonia. In Leydig cells, the amino acid upregulates androgen production by eliciting the adenylate cyclase-cAMP and/or mitogen-activated protein kinase (MAPK) pathways. d-Asp treatment enhances gene and protein expression of enzymes involved in the steroidogenic cascade. d-Asp also directly affects spermatogonial mitotic activity. In spermatogonial GC-1 cells, d-Asp induces phosphorylation of MAPK and AKT serine-threonine kinase proteins, and stimulates expression of proliferating cell nuclear antigen (PCNA) and aurora kinase B (AURKB). Further stimulation of spermatogonial GC-1 cell proliferation might come from estradiol/estrogen receptor β (ESR2) interaction. d-Asp modulates androgen and estrogen levels as well as the expression of their receptors in the rat epididymis by acting on mRNA levels of *Srd5a1* and *Cyp19a1* enzymes, hence suggesting involvement in spermatozoa maturation.

## 1. Introduction

The regulation of steroidogenesis and spermatogenesis involves a complex interaction of a diversity of hormones and intracellular signaling pathways [1,2,3]. Testis steroidogenesis appears to be modulated by a variety of factors in addition to the adenylates. Among these, a considerable interest in the last two decades has been directed to d-aspartate (d-Asp), an amino acid endogenously present in vertebrate testes [4]. With immunocytochemistry, the amino acid has been detected in Leydig cells, Sertoli cells and germ cells, notably in spermatogonia, elongate spermatids and spermatozoa of rat [5], man [6] and lizard [7]. Biochemical studies have demonstrated that in rat testis the highest concentrations of d-Asp are found in testicular venous blood plasma (about 120 nmol/mL), in the rete testis fluid (95 nmol/mL) and epididymal spermatozoa (80 nmol/g wet weight) [8]. Lower concentrations occur in interstitial extracellular fluid, luminal fluid from the seminiferous tubules, and testicular parenchymal cells (11, 23 and 26 nmol/mL, respectively). These values are all higher than peripheral blood plasma levels (6 nmol/mL).

A strong correlation between d-Asp concentration and testosterone (T) levels has been observed in rat testes [9]. At late fetal life, rat testes start synthesizing d-Asp (55 ± 8 nmol/g) and T (200 ± 30 ng/g). After birth, both d-Asp and T concentrations progressively increase, reaching maximum levels at sexual maturity [5,9]. At 80 days after birth, testis levels of d-Asp and T are about 150–200 nmol/g and 380 ± 40 ng/g, respectively [10].

Several studies reported that although exogenous d-Asp administration increased T levels, the effect of the amino acid on the release of other sex steroid hormones may vary between species and between in vitro and in vivo test systems. Both intra-peritoneal (acute treatment; 2.0 µmol d-Asp/g body weight) and oral (chronic treatment; 20 mM d-Asp ad libitum for 15 days) d-Asp administration to adult rats resulted in accumulation of the amino acid in the testis and at the same time induced an increase of serum luteinizing hormone (LH), progesterone (P), and of testis/serum T levels [9,10,11,12,13]. Recent studies have also demonstrated that exogenous d-Asp modulates the expression of both estrogen receptor (ESR) and androgen receptor (AR) in the rat testis [13].

Additionally, in vitro studies have shown that d-Asp may induce T synthesis by directly acting on Leydig cells and/or through the hypothalamus–pituitary–testis axis. In cultured rat Leydig cells d-Asp, alone or in the presence of human chorionic gonadotropin (hCG), upregulates T synthesis by stimulating gene and protein expression of the steroidogenic acute regulatory protein (STAR) [14,15], a transport protein that regulates cholesterol transfer within the mitochondria [16,17]. Experiments on isolated rat hypothalamus demonstrated that d-Asp elicited the release of the gonadotropin releasing hormone, which in turn induced the release of LH from the pituitary gland [10].

Numerous studies carried out in seasonal-breeding vertebrates strongly support a role of d-Asp in endocrine control of reproduction [4]. In both frog, *Pelophylax esculentus* (*P. esculentus*, former *Rana esculenta*), and lizard, *Podarcis s. sicula* (*P. s. sicula*), d-Asp concentration in the testis showed significant variations during the reproductive cycle, with the highest levels in sexually active animals [18,19,20]. Intra-peritoneal injection of d-Asp (2.0 µmol/g body weight) to pre-reproductive and post-reproductive animals increased T levels while 17β-estradiol (E2) levels fell [18,19,20]. Exogenous d-Asp in reproductive frogs, however, increased E2 levels [18,21].

d-Asp-stimulated spermatogenesis has been reported in *P. esculentus* and *P. s. sicula* [7,18]. Recently, in vitro studies on mouse spermatogonial GC-1 cells demonstrated that d-Asp stimulates spermatogonial proliferation [22].

The role played by d-Asp in steroidogenesis and spermatogenesis appears to be mediated by several intracellular signaling pathways. Particularly, there is evidence that in Leydig cells the amino acid modulates steroidogenesis by eliciting the adenylate cyclase-cAMP and/mitogen-activated protein kinase (MAPK; more commonly known as ERK, extracellular signal-regulated kinase) pathways [4]. In spermatogonia, d-Asp may directly activate proliferation through both MAPK and AKT pathways [4,22]. These intracellular pathways could be triggered by binding of d-Asp to the *N*-methyl-d-aspartate receptor (NMDAR) [13]. The complexity of intracellular signal transduction is further increased by potential cross-talk at various steps in the signaling cascades. Here we review intracellular signaling pathways affected by d-Asp in the testis, with focus on Leydig cells and spermatogonia.

## 2. d-Asp and Leydig Cells

Albeit d-Asp specific receptors have not yet been identified, a number of reports indicate that NMDAR has an affinity for d-Asp [23,24,25,26]. NMDAR is assembled as heterologous tetramers comprised of an obligatory GRIN1 subunit (more commonly known as NR1) plus four modulatory GRIN2 subunits (GRIN2A, GRIN2B, GRIN2C, GRIN2D; more commonly known as NR2A, NR2B, NR2C and NR2D, respectively), each encoded by a separate gene [27].

**Figure 1:**
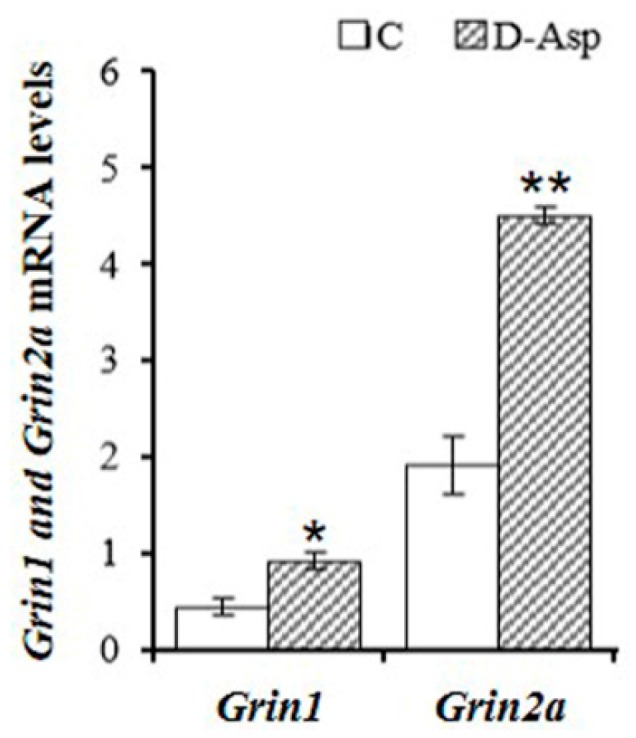
Quantitative Real Time-PCR (qRT-PCR) analysis of NMDAR subunits (*Grin1* and *Grin2A*) in the testis from d-Asp-treated rats (chronic treatment) and controls (C in the figure). Values represent the mean ± SD of five samples. ** *p* < 0.01 and * *p* < 0.05.

*Grin1* and *Grin2A–D* mRNAs are expressed in rat testis [13,28], whereas only *Grin1* and *Grin2B* mRNAs are expressed in mouse testis [29]. Immunohistochemical studies have demonstrated the presence of the GRIN1 subunit in rat Leydig cells [30,31] and spermatogonia [13,30]. At present no study reports the immunolocalization of GRIN2 subunits in the testis. More recently, Santillo and coworkers [13] reported that the expression of both *Grin1* and *Grin2A* mRNAs in rat testes following d-Asp treatment were almost double that of controls (Figure 1).

Further, several pieces of evidence indicate that NMDAR activation induces phosphorylation of MAPK [32]. MAPK is a serine/threonine kinase that occupies a focal point in signal transduction, mainly by activating gene transcription via translocation to the nucleus [33]. NMDAR-dependent MAPK-signaling triggers new gene expression [34]. In the rat testis, d-Asp administration elicited MAPK1 and MAPK3 phosphorylation (P-MAPK1 and 3; more commonly known as P-ERK2 and 1, respectively) [13]. Immunolocalization of P-MAPK 1 or 3 in the interstitium suggests that activation of cAMP and/or MAPK pathways by d-Asp through binding to NMDAR could be involved in steroidogenesis [13]. Previously, cAMP has been implicated as second messenger for the synthesis of T [35,36]. Topo and coworkers [12] reported that rat Leydig cells incubated at 37 °C for 60 min with 10 mM d-Asp showed a five-fold increase of cAMP levels.

**Figure 2:**
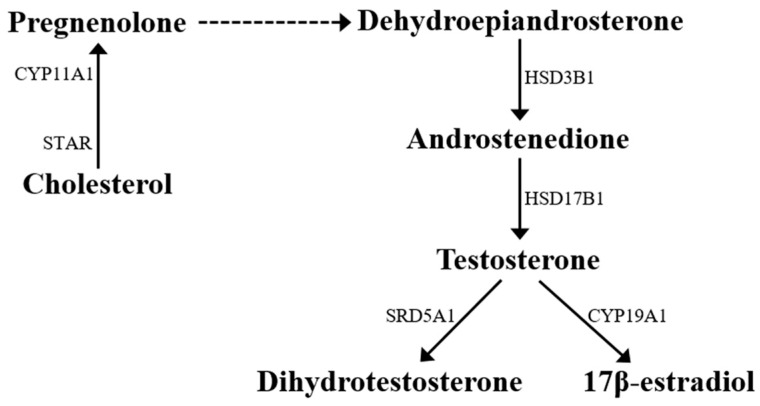
Sex steroid biosynthesis pathway. In Leydig cells cholesterol is translocated by STAR to the inner mitochondrial membrane, where it is converted into pregnenolone by P450 cholesterol side-chain cleavage (CYP11A1). Next in the smooth endoplasmic reticulum, pregnenolone is converted to dehydroepiandrosterone (dashed line) and then androstenedione by 3β-hydroxysteroid dehydrogenase (HSD3B1). Then, 17β-hydroxysteroid dehydrogenase (HSD17B3) catalyzes the conversion of androstenedione to testosterone which finally is converted into 5α-dihydrotestosterone by 5α-reductase (SRD5A1), or into 17β-estradiol by cytochrome P450 aromatase (CYP19A1).

In Leydig cells, steroidogenic pathways begin in the mitochondria with STAR-mediated translocation of cholesterol from intracellular sources (across the outer mitochondrial membrane) to the inner mitochondrial membrane [16,17] (Figure 2). Cholesterol is converted to pregnenolone by the cytochrome P450 cholesterol side-chain cleavage enzyme (CYP11A1; more commonly known as P450scc). Next, in the smooth endoplasmic reticulum, pregnenolone is converted to dehydroepiandrosterone and then androstenedione by 3β-hydroxysteroid dehydrogenase (HSD3B1; more commonly known as 3β-HSD). Then, 17β-hydroxysteroid dehydrogenase (HSD17B3; more commonly known as 17β-HSD) catalyzes the conversion of androstenedione to T (Figure 2).

**Figure 3:**
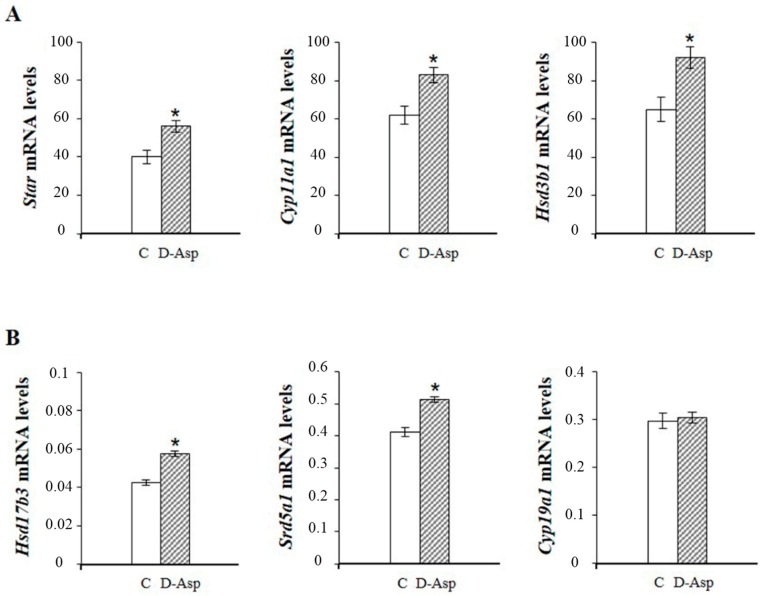
(**A**) qRT-PCR analysis of *Star*, *Cyp11a1* and *Hsd3b1* in the testis from d-Asp-treated rats and controls. The animals were allowed to drink 20 mM d-Asp *ad libitum* for 15 days (chronic treatment); (**B**) qRT-PCR analysis of *Hsd17b3*, *Srd5a1* and *Cyp19a1* in the testis from d-Asp-treated rats and controls. The animals were injected i.p. with 2.0 µmol d-Asp/g body weight (acute treatment). Values represent the mean ± SD of five samples. * *p* < 0.05.

Numerous experimental data show that d-Asp upregulates steroidogenesis at several steps of the steroidogenic cascade. In the frog *P. esculentus*, d-Asp treatment increased *Star* mRNA and protein expression both in reproductive and post-reproductive testis [21]. Further, Raucci and co-workers [37] reported an increase in *Star*, *Cyp11a1* and *Hsd3b1* mRNA levels (Figure 3) and higher concentrations of androstenedione and T in rat testis after chronic d-Asp administration. Accordingly, injection of 2.0 µmol d-Asp/g body weight to sexually mature rats induced an increase of *Hsd17b3* mRNA expression (present data) (Figure 3). Immature Leydig cells cultured with d-Asp showed significantly higher *Star* mRNA and protein levels as well as *Cyp11a1* and *Hsd3b1* mRNA levels relative to controls [37]. d-Asp, in the presence or in the absence of hCG, upregulated T production in rat Leydig cells by increasing the *Star* mRNA and protein expression [14,15,38,39].

In the testis, the main final product of the steroidogenic process is represented by T, which can be converted into either a more potent androgen, 5α-dihydrotestosterone (DHT) by 5α-reductase (SRD5A1; more commonly known as 5α-RED1) or into E2 by cytochrome P450 aromatase (CYP19A1; more commonly known as P450ARO) (Figure 2). In *P. esculentus*, basal mRNA level of *Srd5a1* were significantly higher in reproductive than post-reproductive testes, and levels increased after d-Asp treatment only in the post-reproductive phase [21]. An increase of both *Srd5a1* mRNA and serum DHT have been observed in adult rats 2 h after d-Asp injection (present data) (Figure 3). Numerous studies report that d-Asp is involved in estrogen synthesis through aromatase activity [21,40,41,42,43,44]. In *P. esculentus*, basal mRNA levels of *Cyp19a1* were significantly higher in post-reproductive than reproductive testis [21]. With transition to the reproductive condition, *Cyp19a1* mRNA levels increased by about two-fold in d-Asp-injected testes whereas in the post-reproductive period, no differences were observed [21]. In contrast, in rat testis d-Asp administration was associated with a reduction of CYP19A1 protein levels [13] but not of mRNA (present data) (Figure 3).

It is well known that steroidogenesis in Leydig cells is under strict control by LH binding to its receptor (LHCGR). Recently, Di Nisio and coworkers [39] demonstrated a modulating effect of d-Asp on vesicular trafficking involving LH/hCG-induced internalization of LHCGR and subsequent lysosomal degradation. Most important, a synergism between hCG and d-Asp has been observed.

All the above reports suggest that d-Asp activates spermatogenesis by enhancing T levels. However, recent in vitro experiments indicated that the amino acid directly stimulates spermatogonial proliferation.

## 3. d-Asp and Spermatogonial Proliferation

**Figure 4:**
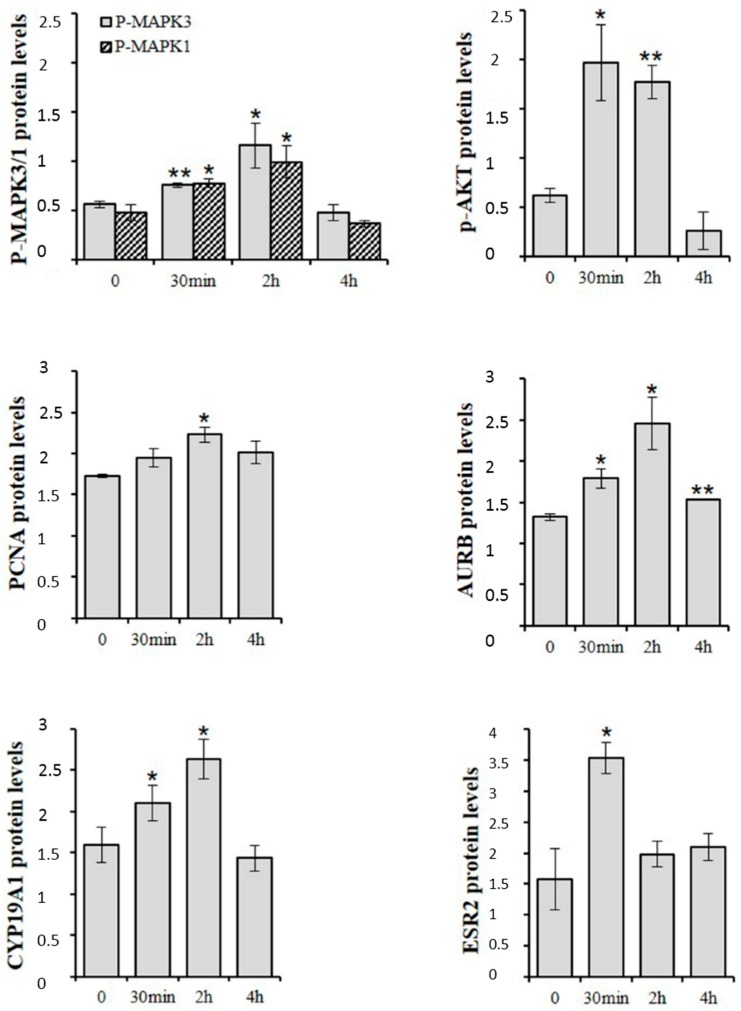
Western blot analysis of P-MAPK3/1, P-AKT, PCNA, AURKB, CYP19A1 and ESR2 in spermatogonial GC-1 cells at various times of d-Asp-treatment. Values represent the mean ± SD of three separate experiments. * *p* < 0.05 and ** *p* < 0.01 versus controls.

d-Asp occurs endogenously in germ cells, particularly in spermatogonia, elongating spermatids and spermatozoa [5,6,7]. Immunohistochemical studies have revealed that in rat testis the GRIN1 subunit of NMDAR and P-MAPK are prevalently localized in spermatogonia [13,30]. In cultured spermatogonial GC-1 cells, exogenous d-Asp induces phosphorylation of MAPK3/1 and AKT proteins [22] (Figure 4). It is known that both MAPK and AKT pathways control several biological functions including gene expression and cell cycle [45] and that they play a crucial role in spermatogonial proliferation [46,47,48]. Interestingly, addition of d-Asp to culture medium of spermatogonial GC-1 cells induced an increase in protein expression of proliferation markers, e.g., proliferating cell nuclear antigen (PCNA) and aurora kinase B (AURKB) (Figure 4). PCNA is a protein expressed in the nucleus of cells in the S phase [49], and AURKB is a key mitotic regulator required for genome stability and for G_2_ to M transition [50,51,52]. Therefore, d-Asp could stimulate spermatogonial proliferation through NMDAR-dependent activation of MAPK and/or AKT pathways, culminating in an increase of PCNA and AURKB protein expression [53]. Strong evidence for d-Asp participation in spermatogonial proliferation has been produced by in vivo studies in low vertebrates. This includes intense immunopositivity for PCNA in the spermatogonia of both *P. esculentus* [18] and *P. s. sicula* [7,19] and an increase of Kit (more commonly known as c-kit receptor) expression in the spermatogonia of *P. s. sicula* following d-Asp treatment [7]. It is well known that stem cell factor/Kitlg (more commonly known as c-kit) signal induces spermatogonial proliferation. In contrast, Tomita and co-workers [54] found a negative effect of d-Asp on the differentiation of premeiotic germ cells.

Nitta and coworkers [55] first demonstrated the presence of a functional CYP19A1 in mouse spermatocytes and spermatids. In the rat, *Cyp19a1* mRNA has been detected in all germ cells, whereas the protein and its activity have been described only in the spermatocytes, spermatids and spermatozoa [56,57]. Spermatogonial GC-1 cells express both mRNA and protein of CYP19A1 [22]. Exogenous d-Asp elicited a rapid increase in CYP19A1 and ESR2 (more commonly known as ERβ) protein expression in spermatogonial GC-1 cells (Figure 4), suggesting d-Asp involvement in the synthesis or activity of E2 in spermatogonia [22]. It has been demonstrated that upon stimulation with E2, the estrogen receptor interacts with signaling proteins such as the tyrosine kinase SRC and PIK3C3 (more commonly known as phosphatidylinositol-4,5-bisphosphate 3-kinase or PI3-K) activating either AKT or a mitogenic pathway that culminates in the activation of MAPK3/1 and lastly the entry into the S-phase [53,58]. On the other hand, estrogen stimulation causes a dose-dependent transient activation of MAPKs in mitotic spermatogonial GC-1 cells [59]. Therefore, d-Asp could potentiate spermatogonial proliferation also through estradiol/ESR2 interaction. However, since ESR1 (more commonly known as ERα) is mainly expressed in spermatogonial GC-1 cells [60] it remains to be demonstrated if d-Asp also acts through this receptor.

## 4. d-Asp and Spermatozoa Maturation

Rat epididymis contains substantial amounts of d-Asp (210–290 nmol/g tissue) and possesses the capacity to take up and accumulate this amino acid if exogenously administered [61]. d-Asp concentration in the rat epididymis and epididymal fluid is about one-eighth and one-third of the testis level, respectively [5]. Spermatozoa produced in the testis enter the caput epididymis, progress to the corpus, and finally reach the cauda region, where they are stored [62]. The main function of the initial segment, caput and corpus of the epididymis is to provide a luminal environment appropriate for the maturation of spermatozoa, whereas the cauda region functions in the storage of mature spermatozoa. T and DHT [63] as well as E2 [64] control the development, structure and function of the epididymis. Falvo and coworkers [61] have demonstrated that in rat epididymis d-Asp modulates androgen and estrogen levels as well as the expression of their own receptors by acting on the expression of *Srd5a1* and *Cyp19a1* genes.

d-Asp also occurs in human seminal plasma and is implicated in male fertility [6]. The treatment of sub-fertile patients with d-Asp improved the number and the motility of the spermatozoa [65].

Recently, Tomita and coworkers [54] have demonstrated that in mouse endogenous d-Asp preferentially accumulates in differentiated spermatids, indicating either transport of d-Asp from other cell type(s) or de novo synthesis in these cells.

## 5. Conclusions

**Figure 5:**
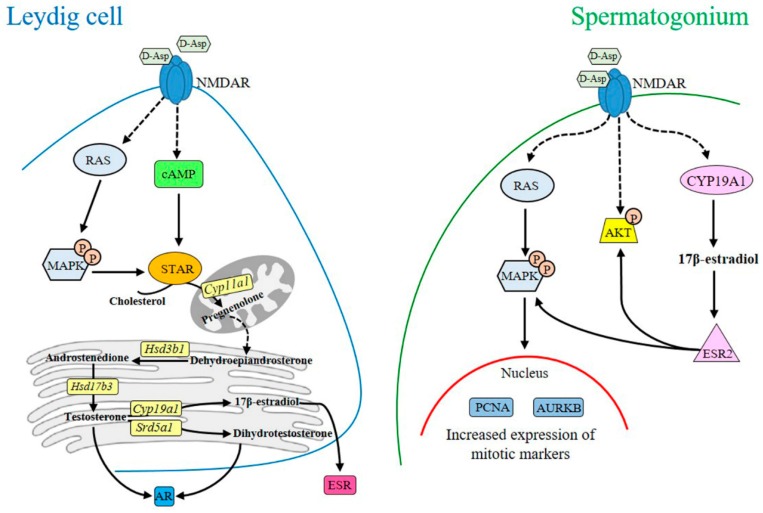
Schematic representation of molecular pathways activated by d-Asp in Leydig cell and spermatogonium. Through the activation of NMDAR, d-Asp up-regulates T production in Leydig cells by increasing STAR expression through cAMP and/or MAPK pathways. STAR regulates cholesterol transfer within the mitochondria. Further d-Asp affects gene expression of *Cyp11a1*, *Hsd3b1*, *Hsd17b3*, *Srd5a1* and *Cyp19a1* enzymes as well as AR and ESR protein expression. In spermatogonial GC-1 cells, d-Asp may directly activate proliferation through MAPK and AKT pathways. Further stimulation of spermatogonial GC-1 cell proliferation could arise from estradiol/ESR2 interaction.

A remarkable body of evidence suggests that d-Asp regulates steroidogenesis and spermatogenesis by eliciting several intracellular pathways possibly via binding to the NMDAR (Figure 5). d-Asp-stimulated gene or protein expression of the steroidogenic cascade enzymes through cAMP and/or MAPK pathways increases both androgen production and expression of androgen receptor (Figure 5). d-Asp may directly activate spermatogonial proliferation through MAPK and AKT pathways (Figure 5). Further, we speculate that stimulation of spermatogonial GC-1 cell proliferation could arise from estradiol/ESR2 interaction. As endogenous d-Asp is known to occur also in more advanced stages of spermatogenesis including spermatocytes and spermatids, future research should be directed to investigate the role of this amino acid in the course of germ cell maturation.

An important role of d-Asp in spermatozoa maturation is also suggested by recent studies demonstrating that d-Asp modulates rat epididymis androgen and estrogen levels as well as the expression of the receptors of these hormones in rat epididymis, by acting on local gene expression of *Srd5a1* and *Cyp19a1* enzymes.
